# Acute and mixed alcohol intoxications in asylum seekers presenting to an urban emergency department in Switzerland

**DOI:** 10.1186/s12889-019-6910-2

**Published:** 2019-05-10

**Authors:** Adam D. Brown, Martin Müller, Trevor Hirschi, Jonathan F. Henssler, Katharina Rönz, Aristomenis K. Exadaktylos, David Srivastava

**Affiliations:** 1 Department of Psychology, New School for Social Research, 80 5th Avenue, New York, NY 10011 USA; 2 Department of Psychiatry, New York University School of Medicine, New York, USA; 3 Department of Emergency Medicine, Inselspital, Bern University Hospital, University of Bern, Bern, Switzerland; 4 Institute of Health Economics and Clinical Epidemiology, University Hospital of Cologne, Cologne, Germany; 5 Department of Psychiatry and Psychotherapy, St. Hedwig Hospital Berlin, Charité University Medicine, Berlin, Germany

**Keywords:** Alcohol disorders, Emergency department, Mixed intoxication, Refugees, Asylum-seeking, Hazardous drinking, Public health, Early intervention

## Abstract

**Background:**

Previous studies have reported an increase in alcohol-and-mixed intoxication (AAMI)-related emergency department (ED) admissions, but less is known about the incidence and characteristics of AAMI admissions to EDs among asylum-seeking patients. Asylum seeking patients may be at higher risk for AAMI due stressors associated with forced migration. The aim of this study was to determine the proportional incidence, population characteristics, and predictors of ED admissions due to AAMI among patients with a residency status of asylum seeker as compared to those with a residency status of Swiss-national.

**Methods:**

This retrospective analysis included all medical consultations from a large, adult ED in Switzerland between January 1, 2013 to December 31, 2016. The residency status of consultations was established if possible, and AAMI was determined utilizing a two-step screening procedure, blinded for residency status. A multivariable logistic regression was performed to determine the odds of AAMI in asylum-seeking patient consultations compared to consultations for Swiss-national patients. In addition, patient characteristics among asylum seekers admitted for AAMI were compared to patients with Swiss-national residency status for AAMI.

**Results:**

In total, 117,716 eligible consultations (Swiss-national patient consultations: *n* = 115,226 and asylum-seeker consultations: *n* = 2490) were included in this study. The proportional incidence of AAMI among asylum seekers was 3.7% (*n* = 92) compared to 1.6% (*n* = 1841) among the Swiss-national patients. AAMI in asylum seekers was associated with higher levels of trauma (37.0% vs. 23.5%, *p* = 0.003), and hospital admission (35.4% vs. 14.1%, *p* < 0.001), but a smaller proportion of chronic alcohol consumption (13.0% vs. 43.5%, *p* < 0.001), and psychiatric referrals (26.1% vs. 49.0%, *p* < 0.001). Multivariable analysis controlling for age, sex, triage category, weekend admission, year of admission, and multiple visits showed a 1.6 times higher odds (95% CI: 1.3, 2.0; *p* < 0.001) for an AAMI-related ED consultation in asylum seeking patients.

**Conclusions:**

These findings show that individuals seeking asylum in a high-income country may be at greater risk for AAMI-related admission than the local population. Given the observed association between AAMI-related ED admissions and trauma, suicidality, and psychiatric referrals among this subpopulation, the data also suggests that co-morbid mental health disorders associated with forced displacement may contribute to hazardous alcohol use.

## Background

Alcohol-related illness appears to lead to higher numbers of Emergency Departments (EDs) admissions. Retrospective studies of medical charts found that up to 28% of alcohol-related ED attendance was associated with alcohol intoxication [1–4]. In rural Australia, studies employing self-report methods to assess alcohol-related ED admissions found that 40% of ED visits were attributed to alcohol use among young adults [5]. Furthermore, in the UK, a weekend sample of EDs revealed that 40–70% of admitted patients had recently consumed alcohol [6]. Relatedly, a prospective study in England found that up to 70% of weekend admissions in the ED were alcohol-related [7].

Cumulatively, these findings suggest that alcohol-related illnesses represent a an area of concern in EDs. However, scant information is known about alcohol-related admissions among vulnerable populations, such as asylum seekers. Given the unprecedented numbers of individuals being displaced globally due to armed conflict, there has been a dramatic rise in the number of individuals migrating and seeking asylum in Europe. A growing body of literature demonstrates the vital role that EDs play in healthcare for asylum seekers. In Europe, asylum seekers are utilizing the ED at high levels [8–10]. Similarly, a recent study examining rates of admission by asylum seekers in an ED in Switzerland found that between 2012 and 2015, the number of asylum seekers admitted to the ED increased by 45%. Within this study, ED admissions were most commonly associated with trauma, infectious diseases, and psychiatric issues [11].

Despite these findings, no studies to date have examined the presence of alcohol in ED admission among asylum seekers. Extant data found that individuals from “strong migratory pressure countries” comprised a greater proportion of ED users and were more likely to receive an alcohol-related diagnosis compared to Italian-born patients [12]. Similarly, non-European born patients accounted for more ED visits than Italian-born patients in Italy, with alcohol being an important factor for ED [13].

The aim of this study was to further elucidate the incidence and characteristics associated with alcohol-related illness among asylum seekers admitted to the ED. Specifically, medical chart data was analysed from a period of over three years, comparing asylum-seeking and Swiss-born patients admitted to a large Swiss ED.

## Methods

**Fig. 1:**
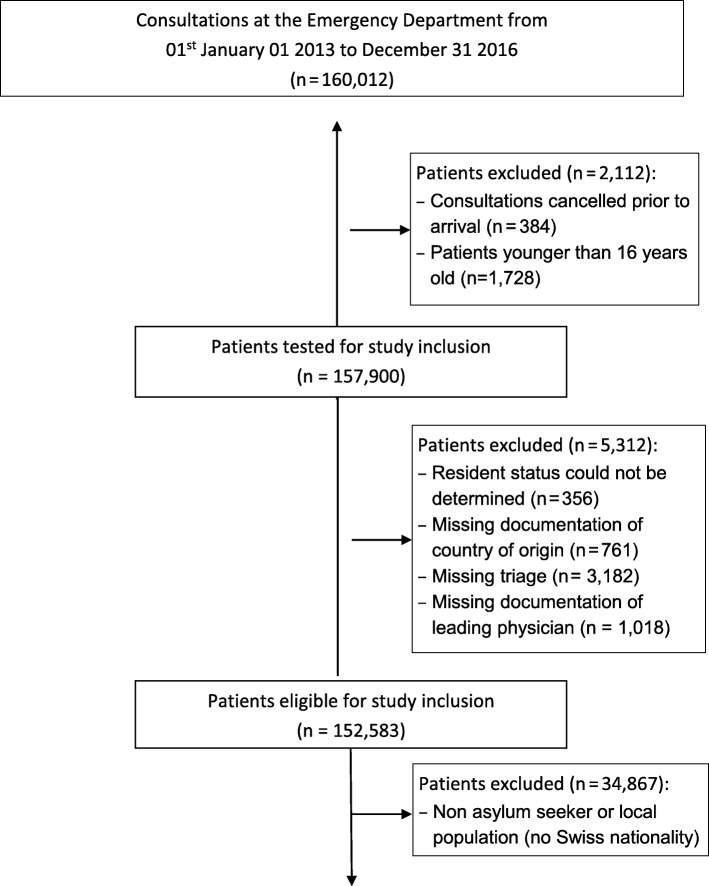
Flow-chart showing the identification of records of patients with asylum seeking and Swiss-national residency status

A retrospective analysis was conducted to identify all adult (≥16 years) patients with a diagnosis (primary or secondary) of acute alcohol intoxication including mixed intoxications (AAMI) with illicit or prescribed drugs who presented to the Bern University Hospital (Inselspital) ED between January 1, 2013 to December 31, 2016. Among all identified patients admitted for AAMI, those whose residency status in the medical chart was entered as “Swiss-national” or “Asylum Seeker” were selected for comparison in these analyses (See Fig. 1).

We obtained the permission of Ethics Committee, within the Canton of Bern (Kantonale Ethikkommission Bern, Ref. No. KEK-BE: 010/2016). The Ethics Committee approved the waiver of consent to analyse these records.

### Data collection and eligibility criteria

Although there is no consensually established cut-off level for blood alcohol concentration to diagnose alcohol intoxication, the criteria in the Diagnostic and Statistical Manual of Mental Disorders-IV (DSM-IV-TR, 2000) was employed for this study. According to the DSM-IV-TR, acute alcohol intoxication refers to the combination of recent alcohol ingestion and concurrent or subsequent changes in behaviour, such as slurred speech and lack of coordination.

Eligible consultations were identified using an unspecific key-word search algorithm – i.e., “intoxication”, “alcohol”, “ethanol”, etc. combined with the Boolean operator “OR” and with different semantic variations to ensure a high sensitivity of the algorithm – in the patients’ ED report from an anonymised, computerised patient database (E-Care, ED 2.1.3.0, Turnhout, Belgium).

All consultations of Asylum-Seeking or Swiss-national adult patients (≥16 years) diagnosed with an acute AAMI were deemed eligible for study inclusion. Patients without a recent primary or secondary diagnosis of acute alcohol intoxication, with duplicate records, with incomplete data sets without documentation of the procedural codes or medical records, and patients whose residency status was neither Asylum Seeker nor Swiss-national were excluded.

### Data extraction

The medical reports of all search results were extracted out of the patient database. After duplicates were removed, the medical history field and the patient diagnosis field were manually screened in full-text for the diagnosis of acute alcohol intoxication.

The following variables were manually coded, and blinded for nationality, upon analysing the medical reports in full-text, or were extracted from the computerised patient database: (a) demographic data such as age, sex, and residency status; (b) breath alcohol concentration, estimated blood alcohol concentration, or other laboratory variables to estimate the blood alcohol concentration, i.e., sodium, potassium, urea, glucoses, and osmolality [14]; (c) signs of mixed intoxication or chronic alcohol abuse; (d) clinical presentation, i.e., any signs of aggression (verbal or physical), vital signs including Glasgow Coma Scale (GCS), trauma grouped by fractures, burns, brain concussion, cerebral or internal bleedings, superficial wounds, contusion, luxation, and others; (e) course at the ED compromise therapy with stiches, rehydration, psychiatrist or special sergeant needed, and sedation, fixation, or intubation performed; and (f) discharge procedure such as emergency surgery, ambulant setting or hospitalisation, as well as initial referral type (walk-in, ambulance etc.).

The study was performed according to Swiss law. As all data was fully anonymised prior to analysis, no consent was needed (Kantonale Ethikkommission Bern, Ref. No. KEK-BE: 010/2016).

### Statistical analysis

Analyses were performed with Stata® 13.1 (StataCorp, College Station, Texas, USA). As normal distribution could not be ensured for most of the variables, all continuous variables were shown as medians with 25th- 75th interquartile ranges (IQR). Categorical variables were shown as percent (absolute number). The Kruskal-Wallis test with the Mann-Whitney-U test as a post-hoc was performed to compare interval variables between different groups. Chi-square test was used to test for an association between categorical variables. The strength of association between being an asylum seeker and presenting with AAMI was quantified by the unadjusted odds ratio accompanied by the 95% Confidence Interval (CI). An adjusted odds ratio was obtained using logistic regression controlling the association for the following covariables: age, sex, triage category, weekend admission, year of admission, and multiple visits.

The final model was altered for sensitivity analysis i) by removing non significant covariables, ii) forcing the variable leading physician in the final model, iii) restricting the analysis to the first visit of a patient, and iv) including all migrant, non-asylum seekers with nationalities other than Swiss (*N* = 34,867) as a category in the final model.

A *p*-value of *p* < 0.05 was considered significant.

## Results

### Patient enrolment and demographic characteristics

Over the course of 36 months, 117,716 patient consultations met inclusion criteria for this study. Among the total number of ED consultations, 2490 were from individuals seeking asylum in Switzerland. Overall, the demographic characteristics of the Swiss-national and asylum-seeking patients differed in a number of ways (See Table 1). Asylum-seeking patients tended to be younger (asylum seeker: median = 29; Swiss-national: median = 52, *p* < 0.001) and male (asylum seeker: % male = 68.3; Swiss-national: % male = 55.6, p < 0.001). In addition, a greater proportion of individuals seeking asylum had multiple visits to the ED (% asylum seeker = 42.3; % Swiss-national = 33.4, p < 0.001), were walk-in admissions (% asylum seeker = 54.6% Swiss-national = 42.3%, *p* < 0.001), seeking care for less acute medical issues (asylum seeker: median = 3; Swiss-national: median = 3, *p* < 0.001), and were more likely to receive ambulatory discharge (% asylum seeker = 68.5; % Swiss-national = 47.7, *p* < 0.001).

**Table 1:** Consultation characteristics

Characteristics	Local population consultation		Asylum seeker consultation		*p*-value	Total	
Number	115,226	(100.0)	2490	(100.0)	–	117,716	(100.0)
Weekend admission, [*n* (%)]	39,045	(33.9)	790	(31.7)	0.024	39,835	(33.8)
Revisit, [*n* (%)]	38,472	(33.4)	1053	(42.3)	< 0.001	39,525	(33.6)
Year of consultation, [*n* (%)]							
2013	25,453	(22.1)	433	(17.4)		25,886	(22.0)
2014	27,477	(23.8)	560	(22.5)		28,037	(23.8)
2015	30,755	(26.7)	669	(26.9)		31,424	(26.7)
2016	31,541	(27.4)	828	(33.3)	< 0.001	32,369	(27.5)
Age, [median (IQR)]	52	(32–69)	29	(22–37)	< 0.001	51	(31–68)
Sex, [*n* (%)]							
Female	51,144	(44.4)	789	(31.7)		51,933	(44.1)
Male	64,082	(55.6)	1701	(68.3)	< 0.001	65,783	(55.9)
Country of origin, [*n* (%)]							
Switzerland	115,226	(100.0)	–			115,226	(97.9)
Eritrea	–		423	(17.0)		423	(0.4)
Syria	–		306	(12.3)		306	(0.3)
Afghanistan	–		293	(11.8)		293	(0.4)
Somalia	–		277	(11.1)		277	(0.2)
Iraq	–		121	(4.9)		121	(0.1)
Other	–		1070	(43.0)	< 0.001	1070	(0.9)
Type of admission, [*n* (%)]							
Walk-in	48,716	(42.3)	1359	(54.6)		50,075	(42.5)
Ambulance	19,282	(16.7)	434	(17.4)		19,716	(16.7)
Previous medical contact (e.g. GP)	18.086	(15.7)	164	(6.6)		18,250	(15.5)
Legal admission	944	(0.8)	98	(3.9)		1042	(0.9)
Other	4484	(3.9)	113	(4.5)		4597	(3.9)
Not specified	23,714	(20.6)	322	(12.9)	< 0.001	24,036	(20.4)
Triage, [median (IQR)]	2	(2–3)	3	(3–3)	< 0.001	3	(2–3)
Leading physician, [*n* (%)]							
Internist	30,600	(26.6)	905	(36.4)		31,505	(26.8)
Surgical	24,728	(21.5)	710	(28.5)		25,438	(21.6)
Psychiatric	5257	(4.6)	40	(1.6)		5297	(4.5)
Specialist	54,641	(47.4)	835	(33.5)	< 0.001	55,476	(47.1)
Discharge, [*n* (%)]							
Discharge at home	54,925	(47.7)	1705	(68.5)		56,630	(48.1)
Hospital admission	41,405	(35.9)	491	(19.7)		41,896	(35.6)
Death	222	(0.19)	1	(0.04)		223	(0.2)
Other	1251	(1.1)	61	(2.5)		1312	(1.1)
Not specified	17,423	(15.1)	232	(9.3)	< 0.001	17,655	(15.0)

### Consultation for acute or mixed-alcohol intoxication

Of the total number of patients admitted to the ED during this time, 1933 patients screened positive for AAMI. Among those who screened positive for intoxication, a greater proportion of patients had asylum-seeking residency status ((% asylum seeker = 3.7; % Swiss-national = 1.6, *p* < 0.001). In addition, those patients screening positive for alcohol intoxication in the ED with asylum-seeking residency status differed from AAMI Swiss-national patients on a number of factors (See Table 2). In terms of demographic characteristics, patients with asylum-seeking residency status were younger (*p* < 0.001) and male (*p* < 0.001). Furthermore, those with asylum-seeking status were more likely to be admitted for trauma (e.g., superficial wounds, *p* < 0.001), less frequently referred to a psychiatrist (*p* < 0.001), more often brought in by police (*p* < 0.001), and less likely to be hospitalized (*p* < 0.001).

**Table 2:** Acute and mixed intoxications characteristics in accordance to the status of residency

Characteristics	Local population consultation		Asylum seeker consultation		*P* value
Number, [*n* (%)]	1841	(100.0)	92	(100.0)	–
Revisit, [*n* (%)]	865	(47.0)	48	(52.2)	0.331
Age, [median (IQR)]	42	(27–53)	29	(23–35)	< 0.001
Sex, [*n* (%)]					
Female	796	(43.2)	6	(6.5)	
Male	1045	(56.8)	86	(93.5)	< 0.001
Triage, [median (IQR)]	3	(2–3)	3	(3–3)	0.092
Mixed intoxication, [*n* (%)]	620	(33.7)	29	(31.5)	0.669
Chronic alcohol abuse, [*n* (%)]	800	(43.5)	12	(13.0)	< 0.001
Suicidal, [*n* (%)]	225	(12.2)	10	(10.9)	0.699
Trauma, [*n* (%)]	432	(23.5)	34	(37.0)	0.003
Superficial wound, [*n* (%)]	261	(14.2)	26	(28.3)	< 0.001
Burns, [*n* (%)]	3	(0.2)	0	(0.0)	0.698
Contusion, [*n* (%)]	97	(5.3)	7	(7.6)	0.332
Luxation, [*n* (%)]	19	(1.0)	0	(0.0)	0.327
Fractures, [*n* (%)]	153	(8.3)	7	(7.6)	0.812
Traumatic brain injury, [*n* (%)]	120	(6.5)	5	(5.4)	0.680
Cerebral bleeding, [*n* (%)]	38	(2.1)	0	(0.0)	0.164
Aggression, [*n* (%)]	121	(6.6)	5	(5.4)	0.666
Fixation needed, [*n* (%)]	11	(0.6)	2	(2.2)	0.071
Police needed, [*n* (%)]	492	(26.7)	38	(41.3)	0.002
Psychiatrist needed, [*n* (%)]	903	(49.0)	24	(26.1)	< 0.001
Special surgery council, [*n* (%)]	175	(9.5)	8	(8.7)	0.796
Stiches performed, [*n* (%)]	209	(11.4)	18	(19.6)	0.017
Sedation performed, [*n* (%)]	105	(5.7)	3	(3.3)	0.319
Intubation performed, [*n* (%)]	23	(1.2)	2	(2.2)	0.444
Operation performed, [*n* (%)]	86	(4.7)	3	(3.3)	0.529
Hospitalization performed, [*n* (%)]	652	(35.4)	13	(14.1)	< 0.001

### Predictors of acute or mixed-alcohol intoxication

A logistic regression was performed to determine if there were differences in the odds of an AAMI-related consultation between patients with asylum-seeking residency status and Swiss-national residency status (See Table 3). In a multivariable analysis controlling for age, sex, triage category, weekend admission, year of admission, and multiple visits, patients in the ED with asylum-seeking residency status, compared to patients with Swiss-national residency status, had a 1.6 times higher odds (95% CI = 1.3, 2.0; *p* < 0.001) of screening positive for an AAMI-related ED. As mentioned, the presence of acute or mixed-alcohol intoxication in the ED was associated with younger age and being male. Patients who screened positive for alcohol intoxication had less acute medical issues (p < 0.001), were more likely to have been seen during the weekend (*p* < 0.05), and had previously been seen in the ED (*p* < 0.001).

**Table 3:** Association of acute and mixed intoxication related emergency consultations (AAMI) and status of residency

AAMI	Odds Ratio (95% CI)	*P*
Unadjusted		
Local population vs. asylum seeker	2.36 (1.89, 2.92)	< 0.001
Multivariable		
Group		
Local population	1.0 (base)	
Asylum seeker	1.58 (1.27, 1.97)	< 0.001
Age [per year]	0.97 (0.97, 0.98)	< 0.001
Sex [being male]	1.10 (1.00, 1.21)	0.043
Triage [from one category to next]	0.59 (0.56, 0.62)	< 0.001
Weekend admission [yes]	1.89 (1.73, 2.07)	< 0.001
Year of admission		
2013	1.0 (base)	
2014	1.02 (0.89, 1.17)	0.769
2015	0.88 (0.77, 1.01)	0.076
2016	1.00 (0.878, 1.42)	0.985
Multiple visit [yes]	2.06 (1.88, 2.26)	< 0.001

### Sensitivity analysis

The final model was altered for sensitivity analysis. The odds ratio only changed slightly after: i) removing non-significant covariables, i.e., year of admission (Adjusted OR = 1.6; 95% CI = 1.3, 2.0; p < 0.001); ii) forcing the variable leading physician in the final model (Adjusted OR = 1.4; 95% CI = 1.1, 1.7; *p* = 0.004); iii) restricting the analysis to the first visit of a patient (Adjusted OR = 1.7; 95% CI = 1.2, 2.3; *p* = 0.001); and iv) including all migrant, non-asylum seekers with nationalities other than Swiss (*N* = 34,867) as a category in the final model (Adjusted OR = 1.6; 95% CI = 1.3, 2.0, *p* < 0.001). The odds of AAMI-related ED consultation were 0.9 times (0.8, 1.0; *p* = 0.047) smaller in the migrant, non-asylum-seeking group compared to the local population.

## Discussion

This is the first study to examine the incidence and referrals of alcohol intoxication-related admissions among asylum seekers in an ED. Compared to Swiss-national patients, asylum-seeking individuals seeking care in the ED were significantly more likely to be admitted for alcohol-related issues. Moreover, trauma and psychiatric issues were the primary complaints among asylum-seeking patients with alcohol-related issues in this sample. These findings converge with related research indicating that individuals who arrive to Europe due to forced migration may have higher rates of mental health concerns, including alcohol abuse [15, 16].

One plausible explanation for the differences observed is that alcohol-related illnesses may represent an important health concern among asylum-seeking patients. There is growing evidence that substance abuse issues are a major public health concern among forcibly displaced persons [17]. However, compared to other diseases, alcohol-related illness among displaced persons and asylum seekers is noticeably understudied by the healthcare community. This represents a major gap in our understanding of the health care needs of a highly vulnerable population, especially considering the well-known medical costs and societal burdens associated with alcohol misuse in general. To date, it has been especially unclear whether hazardous alcohol use has contributed to the increasing numbers of asylum seekers presenting for care in EDs; and, furthermore, relevant demographic and clinical correlates associated with these admissions remain under-examined.

These findings also suggest that the detection and treatment of alcohol-related illnesses among asylum seekers in the ED should be an important priority for hospitals in high-income countries. Given the systemic effects of alcohol on the body, alcohol misuse is associated with a wide range of medical and comorbid mental health conditions and leads to more complex diagnostics and interventions. In addition, studies among forcibly displaced persons have shown that alcohol disorders contribute to non-communicable and communicable (e.g., HIV) diseases [18].

The high numbers of alcohol intoxication-related consultations among this population may seem somewhat counterintuitive, given that the cultural and religious practices associated with the countries-of-origin from which many individuals are seeking asylum are in fact linked to moderation of alcohol use or abstinence. However, exposure to potentially traumatic events and chronic, ongoing stressors, as well as the experience of displacement from one’s family, community and social support systems, are strongly linked with mental health disorders, such as posttraumatic stress disorder (PTSD) and depression, which in turn, have been attributed to increased levels of alcohol use among refugees [15, 18–20]. Findings are consistent with the data from this study showing a strong association between alcohol-related admissions and referrals to psychiatry, e.g., [21]. These findings, along with data from other recent studies (e.g. [18]), underscore the critical importance of assessing for mental health issues during the ED visit in cases in which a patient has asylum-seeking status and presents with alcohol-related issues.

There may be several reasons why trauma was strongly associated with alcohol-related-illness in the asylum-seeker admissions in this study. First, there is considerable research demonstrating that hospital visits related to alcohol are often associated with accidents and injuries [22, 23]. Secondly, it may be possible that individuals with asylum-seeking status are more likely to be brought to the ED whereas Swiss-national patients may be more likely to seek specialist care for similar injuries. The greater incidence of trauma is unlikely to be associated with disruptive behaviour among the asylum-seeking patients, as there were no differences in levels of aggression between the two groups. Further investigation is warranted to examine the factors underlying the high rates of trauma admission and how these factors may impact treatment decisions and quality of care.

Several limitations must be acknowledged. Firstly, this study was a retrospective analysis using medical charts. Future work would benefit from patient interviews and additional self-report measures. Additionally, there was limited patient data in the charts pertaining specifically to alcohol misuse, and information relating to additional factors that may have contributed to hazardous alcohol use (e,g., specific types of mental health disorders, socioeconomic status) was largely unavailable. Lastly, although this study was conducted in a large metropolitan university hospital, it is unclear if these findings will generalize to other EDs. It will be important for future studies to examine if these results can be validated in other hospitals.

## Conclusions

Notwithstanding the aforementioned limitations, these findings offer strong support that it is incumbent upon the healthcare community to more actively and effectively engage in the prevention, screening, and intervention of alcohol-related illness among asylum seekers. Furthermore, given the increased utilization of EDs among asylum seekers in general (e.g. [11]), these data suggest that EDs may serve as critical sites for the development and implementation of alcohol disorder detection programs and the dissemination of public health campaigns. In addition, these results echo recent calls made by others [17], alerting us to critical gaps in care and the necessity of focusing more attention toward the negative impacts of alcohol use on refugees globally. If efforts to combat this public health issue are to achieve success, they will require coordinated research and program development across humanitarian agencies, non-governmental organizations, governmental bodies, and research communities [17], and the data collected from EDs may function as an important aid in guiding effective and culturally competent interventions, as well as their subsequent dissemination.

## Abbreviations

AAMI: Alcohol Abuse and Mixed Intoxication
DSM: Diagnostic and Statistical Manual of Mental Disorders
ED: Emergency Department
